# Enhancement of Mechanical Properties and Rolling Formability in AZ91 Alloy by RD-ECAP Processing

**DOI:** 10.3390/ma12213503

**Published:** 2019-10-25

**Authors:** Qiong Xu, Aibin Ma, Yuhua Li, Bassiouny Saleh, Yuchun Yuan, Jinghua Jiang, Chaoying Ni

**Affiliations:** 1 College of Mechanics and Materials, Hohai University, Nanjing 211100, China; 15251856392@163.com (Q.X.); sduhua@126.com (Y.L.); bassiouny.saleh@hhu.edu.cn (B.S.); yychehai@163.com (Y.Y.); jinghua-jiang@hhu.edu.cn (J.J.); 2 Department of Materials Science & Engineering, University of Delaware, Newark, DE 19716, USA; 3 Suqian Institute of Hohai University, Suqian 223800, China

**Keywords:** AZ91 alloy, RD-ECAP processing, post-ECAP rolling, rolling formability, mechanical properties

## Abstract

In this study, the influence of rotary-die equal channel angular pressing (RD-ECAP) processing on the mechanical properties and rolling formability of AZ91 alloys was investigated. The as-cast and pre-homogenized AZ91 alloys were pre-processed by RD-ECAP for 16 passes at 573 K and subjected to post-ECAP rolling at 573 K with a rolling speed of 10 m/min. The microstructure and deformation characteristics of the AZ91 alloys were characterized. Results demonstrated that fine-grained AZ91 alloys with improved strength and ductility were obtained via the high-pass RD-ECAP processing, indicating a good plastic formability. The ECAP-ed alloys were easily rolled at 573 K from 4.5 mm to 1.1 mm in thickness without edge cracking. After rolling, heterogeneous grain structures were observed with large numbers of twins and shear bands that created strong basal textures. The rolled AZ91 alloys exhibited higher tensile strength and appropriate elongation. The post-ECAP rolling was successfully used in the high productivity of AZ91 rolled plates with good mechanical properties.

## 1. Introduction

In recent years, Mg alloys have become appealing candidates for the electronics, automotive and aviation sectors as the lightest metallic material in structural applications [1,2,3]. Mg-9Al-Zn (AZ91) alloys have received increasingly attention among different Mg alloys due to their comparatively elevated strength, adequate resistance to corrosion, sound damping capacity and good machinability [4,5]. However, due to the HCP structure and abundant dendritic second phases resulted from the high Al content, the formability and ductility of AZ91 alloys are poor [6,7,8]. Plastic deformation (especially rolling) is difficult to conduct on AZ91 alloys. In particular, edge cracking occurs during rolling, and after rolling ductility is generally insufficient, causing the product quality and productivity to be significantly lowered. For this reason, high performance rolled AZ91 alloys with minimum material loss are particularly important to be developed in order to precipitate industries.

The deformation mechanism of Mg alloys depends on a combination of grain size and crystallographic orientation, in which texture plays a very important role. The traditional rolling process typically produces a strong texture along rolling direction with a limited deformation capacity, making it difficult for the rolling Mg alloys to achieve high deformations [9,10]. Some researchers have developed new rolling techniques such as asymmetric rolling (ASR) [11,12], high-ratio differential speed rolling (HRDSR) [13,14] and hard-plate rolling (HPR) [15,16] to get AZ91 alloy rolled sheets. However, most of these studies on rolling AZ91 alloys were not sufficiently described with macroscopic opinions, i.e., the edge crack reduction or elimination of the finishing products. In addition, these newly established methods require greater manipulation or offer restricted access to experimental research and/or factory manufacturing, as opposed to the mature traditional rolling method.

The promising way to precipitate conventional rolling on AZ91 alloy is to pre-modified the structure and change the formability of the alloy before rolling. Grain refinement by severe plastic deformation (SPD) is a promising direction for improving the formability of Mg alloys [17,18,19,20]. The equal channel angular pressing (ECAP) is currently the most advanced SPD process for producing massive ultra-dense grained metallic materials which are potentially suitable for practical applications [19,21,22,23]. This method was first introduced in the 1980s by Segal and his co-workers and developed in the 1990s as an important SPD approach in the preparation of fine-grained materials by Valiev et al. [24,25,26]. Studies have found that the ECAP process can contribute to a specific texture with high Schmidt factors with the cooperation of grain refinement and second phase homogenization [27,28,29]. These ECAP-ed structures demonstrate the mechanical advantages of enhanced strength and ductility at room temperature and superplasticity at elevated temperatures. Therefore, an interesting attempt is to examine the effect of rolling after ECAP on the microstructure and mechanical properties of ECAP-ed alloys. Yuan and Lu et al [30,31] have successfully applied post-ECAP cold rolling on ZK60 and Mg-Gd-Zn-Zr alloys and achieved a good combination of high strength and good ductility. This indicates a feasibility of manufacturing AZ91 rolled sheets or plates with good mechanical properties and high productivity through a combined process of ECAP and conventional rolling.

For conventional ECAP processing, the pressed billet must be removed from the die and re-inserted into the die for the next pass, often after which the billet should be re-heated and re-machined to fit the channel size. A lot of time is required to achieve the desired microstructure and part of the samples will be lost, which makes it difficult to implement on an industrial scale. To overcome this difficulty, Nishida et al. [32] developed a new ECAP process for solving this problem using rotating die, namely rotary-die equal channel angular pressing (RD-ECAP). This RD-ECAP equipment can achieve high ECAP deformation passes with one-time loading and unloading, which can protect samples and improve efficiency. Our research group has applied RD-ECAP on various materials to achieve grain refinement and property improvement [23,33,34,35,36,37]. Recently, up-scaled RD-ECAP equipment was developed and put into use (sample dimension of 50 mm × 50 mm × 100 mm). Therefore, the current work is to conduct a high-pass RD-ECAP pre-processing on AZ91 alloys and then apply multi-step post-ECAP rolling on the ECAP-ed alloys. Investigations focus on the effect of ECAP pre-processing and post-ECAP rolling on the microstructure and mechanical property of AZ91 alloys, especially the effect of ECAP pre-processing on the rolling formability of the alloy. The present work is of great significance for the further development of rolling Mg alloys, and provides reference for the study of deformation behavior of highly deformed Mg alloys.

## 2. Experimental Methods

**Figure 1:**
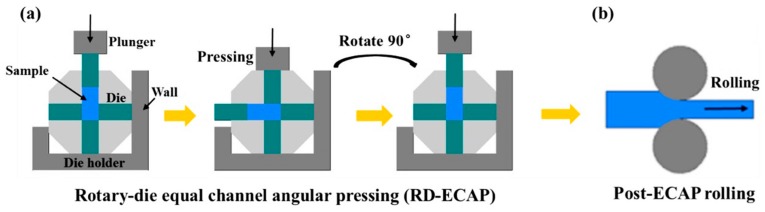
Processing schematic of (**a**) RD-ECAP and (**b**) post-ECAP rolling.

In the current work, an up-scaled RD-ECAP equipment (Wuxi Haofei Machinery Factory, Wuxi, Jiangsu, China) was used to prepare industrial-scale ECAP samples (50 mm × 50 mm × 100 mm), and a two-roll mill device (Φ 240 mm × 400 mm) was used for the rolling processing. Figure 1 illustrates the schematics of the sample processing route. Up-scaled bulk samples with dimensions of 50 mm × 50 mm × 100 mm were machined from a commercial AZ91 alloy ingot. Some bulk samples were homogenized at 693 K for 20 h and then quenched in water. Then the as-cast and as-homogenized bulk samples were processed at 573 K for 16 passes through a 90° RD-ECAP die. As shown in Figure 1a, the channel angle of the die was 90° and the curvature angle was 0°. In the initial state, the sample was placed in the vertical channel and would be extruded from the left horizontal channel. Then, the hexagon mold would be rotated clockwise 90° back to the initial position for the next pass extrusion. Therefore, multiple passes of ECAP can be carried out on the samples rapidly and easily. More details of the operation principle can be found in earlier works [38,39,40]. After ECAP, plate samples with dimensions of 50 mm × 50 mm × 4.5 mm) were cut from the center of the ECAP-ed billets along the longitudinal direction. Plate samples with the same dimensions were also cut from the as-cast and as-homogenized alloys for rolling. The rolling process was performed at 573 K with a rolling speed of 10 m/min. During hot rolling, the samples were reheated in a furnace and kept for 10 min prior to each rolling pass. The reduction per rolling pass was ~10%. Rolling samples were rolled to the final thickness of 1.1 mm with a total thickness reduction of ~75%. Finally, samples of eight different manufacturing strategies were collected for studies with the original as-cast material marked as ‘C sample’, the as-cast sample after homogenization marked as ‘H sample’, ECAP-ed samples processed from ‘C sample’ and ‘H sample’ are marked as ‘C-ECAP sample’ and ‘H-ECAP sample’, respectively. The rolled samples processed from ‘C sample’, ‘H sample’, ‘C-ECAP sample’ and ‘H-ECAP sample’ are marked as ‘C + R sample’, ‘H + R sample’, ‘C-ECAP + R sample’ and ‘H-ECAP + R sample’, respectively. Table 1 lists all of the samples.

Tensile tests were performed with a universal tensile testing machine (UTM4294X, Suns Technology Stock Co. LTD, Guangming, Shenzhen, China) at a constant speed of 0.5 mm/min. Dog-bone geometry tensile specimens were cut along the planes that coincided with extrusion direction and rolling direction, with a gauge size of 2 mm × 2 mm × 6 mm. Three tensile samples were tested and the median strength and ductility values were determined. The Olympus BX51M optical microscopy (Olympus BX51M, Shinjuku, Tokyo, Japan) was used for the overall microstructure observations. TEM analysis was made with a FEI Tecnai-G2 thermo-emission transmission electron microscope (FEI-Tecnai G2, Hillsboro, OR, USA) at an accelerating voltage of 200 kV. The electron back-scatted diffraction (EBSD) analysis was conducted on SEM (Hitachi S-3400N, Hitachi, Chiyoda, Tokyo, Japan) equipped with a HKL-EBSD system. The texture and phase analysis were carried out by means of X-ray diffractometer (XRD, Bruker D8, Bruker, Billerica, MA, USA) with Cu Kα radiation.

## 3. Results and Discussion

### 3.1. Microstructures of As-cast and As-homogenized AZ91 Alloys

**Figure 2:**
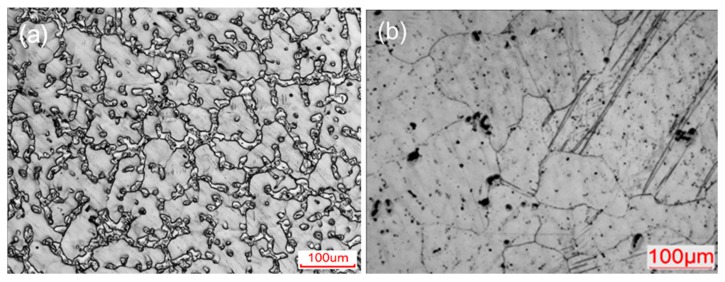
Optical micrographs of (**a**) as-cast and (**b**) as-homogenized AZ91 alloys.

Figure 2 shows the optical micrographs of the as-cast and as-homogenized alloys, i.e., the C sample and H sample. Microstructure in Figure 2a shows a typical dendrite structure of primary α-Mg with an average size of ~100 μm separated by networks of β-Mg_17_Al_12_ precipitates in the C sample. After homogenization at 693 K for 20 h (i.e., H sample), almost all of the β-Mg_17_Al_12_ precipitates are dissolved into the α-Mg matrix, and the matrix grains grow up because of the long-term homogenization in the high temperature. As a result, the H sample demonstrates clear grain boundaries with considerably decreased second phases, and form a homogenized microstructure with coarse grains of ~220 μm, as shown in Figure 2b.

### 3.2. Microstructures of ECAP-ed AZ91 Alloys

**Figure 3:**
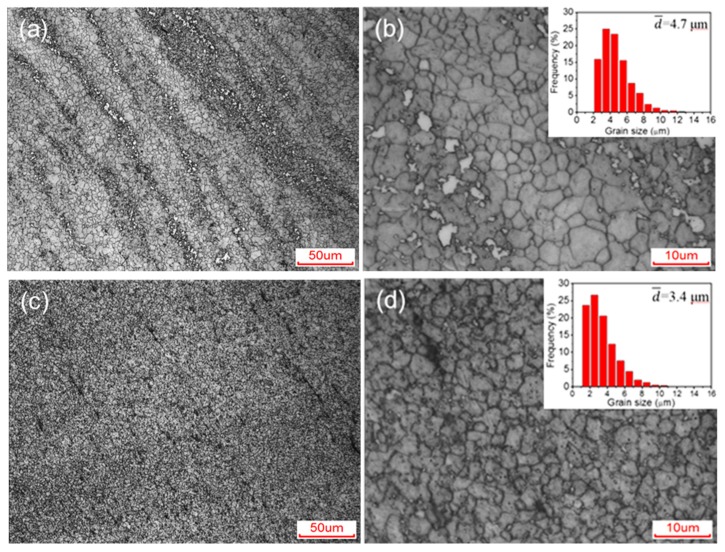
Optical micrographs of (**a**,**b**) C-ECAP and (**c**,**d**) H-ECAP AZ91 alloys in (**a**,**c**) low-magnification and (**b**,**d**) high-magnification.

Figure 3 shows the optical micrographs of the C-ECAP and H-ECAP samples. After the ECAP processing, the grain sizes of α-Mg are remarkably refined and homogenized in both samples as dynamic recrystallization (DRX) occur during the pressing. Interestingly, the grain sizes in these two samples are comparable, while the overall microstructures are not the same. Figure 3a,b show that both the α-Mg matrix and the coarse β-Mg_17_Al_12_ phases in the as-cast sample (i.e., C sample) are remarkably refined and homogenized after 16 passes of RD-ECAP processing (i.e., C-ECAP sample). The original coarse β-Mg_17_Al_12_ networks in Figure 2a are reformed into finer banding distributions in Figure 3a, which highlights the shear deformation flow line of the RD-ECAP processing. As shown in Figure 3b, the matrix grains are refined to sizes ranging from ~2 μm to ~10 μm with an average size value of ~4.7 μm; finer second phases dispersed between the matrix grains in the banding areas. In addition to the difference of the overall structure described above, it also can be detected that the proportion of β-Mg_17_Al_12_ phases in the C-ECAP sample is lower than that in the C sample, which implies solid solution occurred during the RD-ECAP processing. The H-ECAP sample shows a different microstructure from C-ECAP sample, as shown in Figure 3c,d. A more refined and homogenized structure is formed in the H-ECAP alloy. Most grains are refined to sizes ranging from ~1μm to ~8 μm, with an average size value ~3.4 μm, as shown in Figure 3d. Tiny second phases can be carefully detected around the grain boundaries in the whole version in Figure 3d, implying second phase precipitation happened during the RD-ECAP processing. 

**Figure 4:**
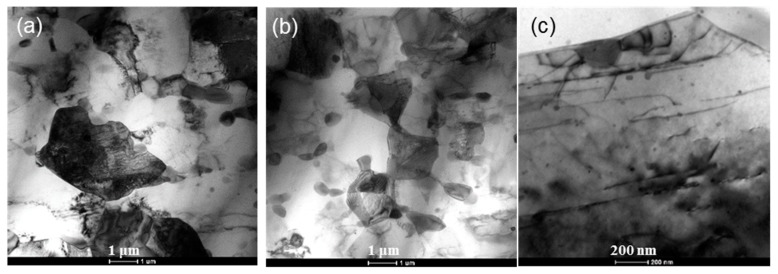
TEM micrographs of H-ECAP AZ91 alloy: (**a**,**b**) matrix grains surrounded by small-size β-Mg_17_Al_12_ precipitates; (**c**) dislocations and nano-sized β-Mg_17_Al_12_ precipitates inner the matrix grain.

TEM photographs in Figure 4 further illustrate more microstructure details of H-ECAP alloy. Figure 4a and b clearly show the matrix grains with sizes of ~1 μm to ~3 μm surrounded by small-size β-Mg_17_Al_12_ precipitates with sizes of ~200 nm to ~500 nm. As the H-ECAP sample is processed from the H sample, in which there is a high concentration of solid solution elements, the second phases precipitate out during the ECAP processing. Most of these second phase precipitates disperse along the grain boundaries and effectively pin the grain boundaries and impede the grain growth, thus to form finer matrix gains. High density of dislocations can be observed in some matrix grains (black matrix grains); nano-sized second phases are also observed inner the matrix grain, as shown in Figure 4c.

### 3.3. Characteristics of Rolled AZ91 Alloys

**Figure 5:**
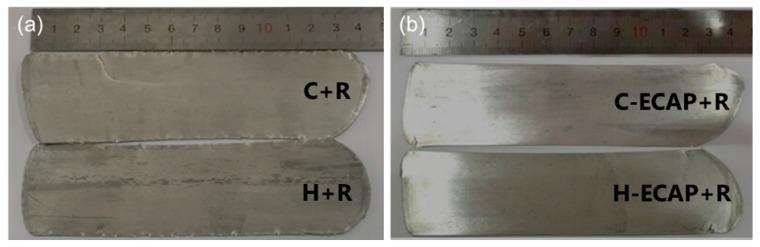
Macroscopic views of (**a**) C + R and H + R rolled alloys; (**b**) C-ECAP + R and H-ECAP + R AZ91 rolled alloys.

Rolling process was applied on the C, H, C-ECAP and H-ECAP samples to get C + R, H + R, C-ECAP + R and H-ECAP + R rolled plates, respectively, as described in the experimental part. The macroscopic views of the rolled AZ91 plates are shown in Figure 5. Obvious edge cracks are observed in the C + R and H + R samples (obtained after direct rolling process on the C and H samples), as shown in Figure 5a. There are almost no cracks in the edge of C-ECAP + R and H-ECAP + R AZ91 plates (obtained by rolling process on the C-ECAP and H-ECAP samples, i.e., post-ECAP rolling), as shown in Figure 5b. This phenomenon implies the RD-ECAP processing has a significant influence on the rolling formability of the AZ91 alloys.

**Figure 6:**
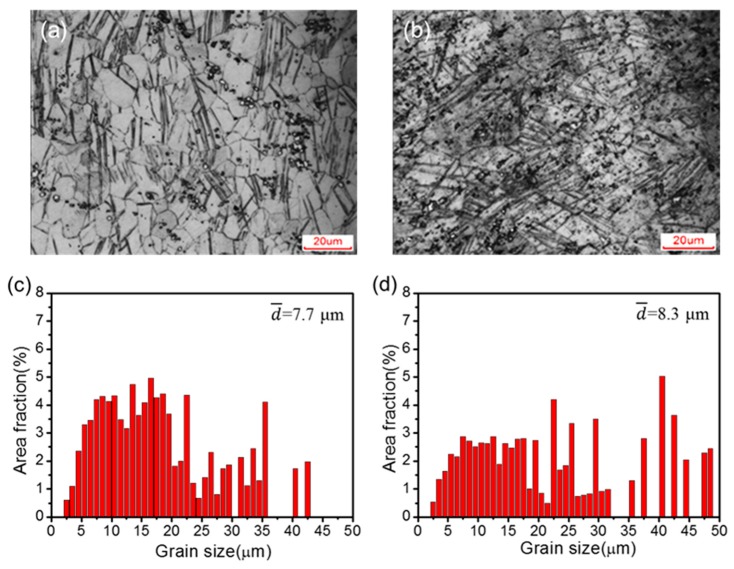
(**a**,**b**) Optical micrographs and (**c**,**d**) grain area distributions of (**a**,**c**) C-ECAP + R rolled alloy and (**b**,**d**) H-ECAP + R rolled alloy.

Figure 6 shows the optical microstructure and the grain area distributions of the C-ECAP + R and H-ECAP + R rolled alloys. It can be seen that the post-ECAP rolling contributed to heterogeneous microstructures with large amount twins and shear bands in both samples. The fine grains yield from ECAP processing illustrated in Figure 3 and Figure 4 are reformed into coarse ones with wide grain size ranges, as shown in Figure 6c,d. The average grain size of C-ECAP + R and H-ECAP + R sample is increased to ~7.7 μm and ~8.3 μm, respectively. These grain sizes are listed in Table 2 and compared with those of the C, H, C-ECAP and H-ECAP samples. The grain sizes of the C-ECAP + R and H-ECAP + R rolled alloys are coarsen a little bit compared to those of the C-ECAP (~4.7 μm) and H-ECAP (~3.4 μm) alloys, while they are significantly refined compared to those of the C (~100 μm) and H (~220 μm) samples. Apart from the grain size change, different changes happened in the amount and morphology of β-Mg_17_Al_12_ phases after the post-ECAP rolling. Compare to the C-ECAP sample (Figure 3a,b), smaller amount of fine second phase particles remain in the C-ECAP + R sample (Figure 6a), and the remained second phases have a distribution tendency along the rolling direction, while compared to the H-ECAP sample (Figure 3c,d), the β-Mg_17_Al_12_ phases are coarsened and accumulated in the H-ECAP + R sample (Figure 6b) and have no obvious distribution tendency.

**Figure 7:**
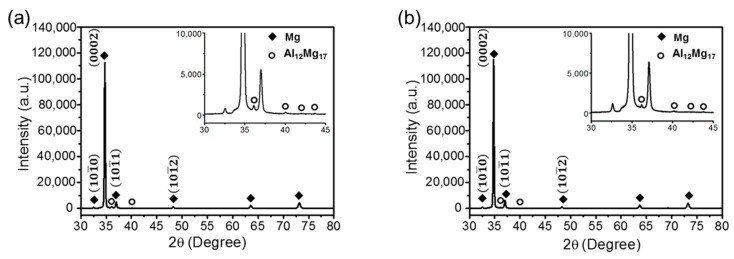
XRD patterns of (**a**) C-ECAP+R and (**b**) H-ECAP+R AZ91 rolled alloys.

Figure 7 shows the X-ray diffraction (XRD) patterns of the C-ECAP + R and H-ECAP + R rolled alloys. According to Figure 7a,b, there is a strong (0002) peak in each of the XRD patterns, indicating that both of the rolled samples exhibit a strong basal texture after rolling. Small ($10\bar{1}0$) and ($10\bar{1}1$) peaks are also observed and the level of the intensity are almost similar to each other. In general, a basal texture was observed for magnesium alloys with a HCP structure because the critical resolved shear stress (CRSSs) of the basic slip is considerably smaller than that of the non-basal slip [10]. The β-Mg_17_Al_12_ phase peaks can be detected from the insets in Figure 7a,b, which present the enlarged XRD patterns between 30° ~ 45°.

### 3.4. Mechanical Properties

**Figure 8:**
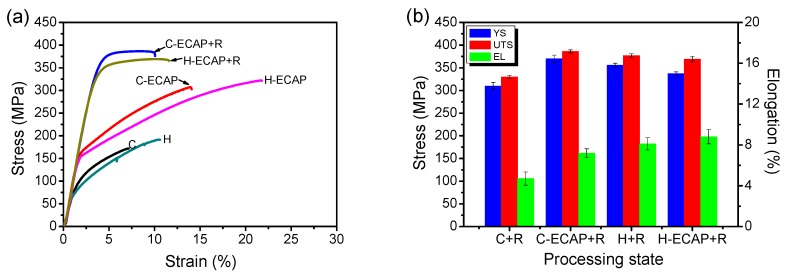
(**a**) Typical tress-strain curves of AZ91 alloys at different processing states; (**b**) the mechanical properties of C + R, C-ECAP + R, H +R and H-ECAP + R rolled samples.

Figure 8 shows the typical tensile stress-strain results of AZ91 alloys after different processing states. As shown in Figure 8a, the C and H samples show relatively poor mechanical properties with ultimate tensile strengths (UST) under 200 MPa and strain less than 6% and 10%, respectively. After 16 passes of ECAP, the UTS and ductility increase remarkably in both ECAP-ed alloys, i.e., the C-ECAP and H-ECAP samples. The UTS of the C-ECAP and H-ECAP sample increase to 307 MPa and 322 MPa, respectively; the elongations (EL) increase to ~11.5% and ~19.6%, respectively. These present results demonstrate that the as-homogenized alloy yields more excellent mechanical properties than the as-cast alloy when subjected to RD-ECAP processing in this work. However, despite the improvement in the UTS and EL, the yield strengths (YS) are still low in both ECAP-ed alloys (<200 MPa). After post-ECAP rolling process, both of the UTS and YS are remarkably increased in these two rolled alloys, i.e., the C-ECAP + R and H-ECAP + R samples, of which the UTS are increased to 386 MPa and 366 MPa, respectively; and the YS are increased to 370 MPa and 337 MPa, respectively. In addition to the increased UTS and YS, the rolled alloys possess moderate ductility with elongation of ~7.2% and ~8.8%, respectively, which resemble the findings obtained by other researchers [41,42]. Indeed, some evidence on moderate elongation can also be found in Figure 5. The specimens of C-ECAP + R and H-ECAP + R in conjunction with the macroscopic views in Figure 5 have smooth surfaces and a few edge cracks, which leads to better plastic deformation and ductility.

The mechanical properties of the C-ECAP + R and H-ECAP + R rolled samples are compared to C+R and H+R samples in Figure 8b. Compare the C + R and C-ECAP + R samples, the C-ECAP + R sample exhibits higher UTS, YS and EL, which means the ECAP pre-processing has a positive effect on the mechanical properties of the rolled AZ91 alloy without pre-homogenization. Compare the H + R and the H-ECAP + R samples, the mechanical properties are very close to each other, with a slight increase in the EL and a slight decrease in YS and UTS, which illustrates that the ECAP pre-processing has little improvement on the mechanical properties of the rolled AZ91 alloy with pre-homogenization. However, from the macroscopic view shown in Figure 5, the improvement on the edge cracking contributed by the ECAP pre-processing is obvious and significant. If compare all of the rolled alloys, it can be seen that the C-ECAP + R exhibit highest UTS and YS, although the EL is a little lower than the H + R and H-ECAP + R samples. Considering the time-consuming and energy-consuming homogenization, applying a post-ECAP rolling on the C-ECAP alloy without pre-homogenization is an effective way to obtain high-strength and moderate-ductility AZ91 Mg rolled sheets with improved edge cracks.

### 3.5. Discussion

**Figure 9:**
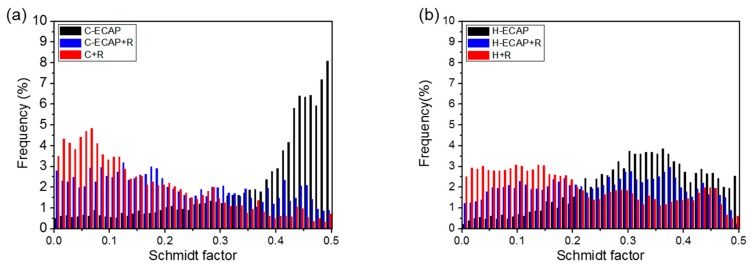
Schmidt factor statistics of (**a**) C-ECAP, C-ECAP + R and C + R alloys; (**b**) H-ECAP, H-ECAP + R and H + R alloys.

The present results show that the high-strength and moderate-ductility AZ91 Mg rolled sheets with improved edge cracks can be obtained by applying post-ECAP rolling on the ECAP-ed AZ91 alloys. However, when applied rolling directly on the as-cast and as-homogenized alloys, serious edge cracking occurred (Figure 5), although the UTS and YS are still increased (Figure 8). This phenomenon implies the RD-ECAP processing greatly changed the structure inner the materials and provided favorable conditions for subsequent rolling. Microstructure characterizations in Figure 3 and Figure 4 have shown that homogeneous refined microstructures were formed in ECAP-ed samples, with second phase refinement and homogenization. Despite grain size and second phases, crystallographic orientation is another important factor affecting the mechanical properties and formability of the Mg alloys [12,27,28]. It was reported that grains with higher Schmidt factors are initially found in the orientations where deformation is easier to occur [28,43]. As shown in Figure 9, the Schmidt factor statistics show significant differences in ECAP-ed and rolled alloys. From both histogram graphs, we can see that both of the ECAP-ed samples, i.e., C-ECAP and H-ECAP samples exhibit higher Schmidt factors with an average value of 0.37 and 0.32, respectively. The samples directly processed by rolling, i.e., the C + R and H + R samples exhibit lowest Schmidt factors with an average value of 0.16 and 0.20, respectively. The alloys processed by ECAP and post-ECAP rolling, i.e., C-ECAP + R and H-ECAP + R samples show middle Schmidt factors, forming orders of C-ECAP > C-ECAP + R > C + R and H-ECAP > H-ECAP + R > H + R. This illustrate that the ECAP processing contributes to more gains with higher Schmidt factors which are favorable for further rolling deformation, and the combined method of ECAP and post-ECAP rolling could achieve better ductility than single rolling.

As illustrated in Figure 8b, the C-ECAP + R sample shows higher mechanical properties than the C + R sample, while the H + ECAP + R and H + R samples exhibit similar mechanical properties. This means that although the ECAP processing contributes to finer grain structure and higher mechanical properties in the pre-homogenized alloy (Figure 3 and Figure 8a), when applying both ECAP and post-ECAP rolling on the alloys, initial pre-homogenization is not encouraged. The C-ECAP + R sample has highest UTS of 386 MPa and YS of 370 MPa, and the EL is comparable to H + R and H-ECAP + R samples (~7.2% compare to ~8.1% and ~8.8%). This phenomenon implies that with the high-pass ECAP and rolling processing, the time-consuming and energy-consuming homogenization could be skipped, that is to say, applying ECAP and rolling on the as-cast sample can achieve high-performance and reduced edge cracks in the alloy, which provides great convenience for practical production and applications.

## 4. Conclusions

In this study, high-performance AZ91 alloy rolled plates with reduced edge cracks were successfully processed by RD-ECAP and post-ECAP rolling from as-cast and as-homogenized alloys with a thickness reduction of ~75 %. Microstructure, mechanical properties and rolling formability of AZ91 alloy subjected to the multi-pass RD-ECAP were investigated. From this study, the following conclusions can be drawn:

(1) The high-pass RD-ECAP process provides an effective procedure for grain refinement and homogeneity of β-Mg_17_Al_12_ phases in AZ91 alloys. The as-cast and as-homogenized alloys after ECAP at 573 K for 16 passes exhibit average grain size of ~4.7 μm and ~3.4 μm, respectively. The fine-grained structures with high Schmidt factors formed by ECAP process contribute to improved strength and ductility, and the significant formability improvement in the post-ECAP rolling.

(2) After rolling, heterogeneous structures with coarse grains and strong basal textures are formed. The UTS and YS after rolling were effectively improved. Post-rolling on the ECAP-ed alloys can effectively reduce and/or eliminate edge cracking and achieve moderate ductility, while direct rolling on the as-cast and as-homogenized alloys resulted in obvious edge cracks. 

(3) The combined process of RD-ECAP and post-rolling processing can achieve strength enhancement and suitable elongation in AZ91 alloys with improved edge cracking, and offer a possibility of skipping the time-consuming and energy-consuming homogenization treatment, making the fabrication of AZ91 sheets with good mechanical properties and high productivity possible.

## Figures and Tables

**Table 1:** Abbreviations for samples used in this work.

Abbreviation	State
C	As-cast
H	Homogenization
C-ECAP	As-cast and ECAP
H-ECAP	Homogenization and ECAP
C+R	As-cast and rolling
H+R	Homogenization and rolling
C-ECAP+R	As-cast and ECAP and rolling
H-ECAP+R	Homogenization and ECAP and rolling

**Table 2:** Estimated average grain sizes of AZ91 alloys at different processing states.

Processing State	C	H	C-ECAP	H-ECAP	C-ECAP + R	H-ECAP + R
Average grain size (μm)	~100	~220	~4.7	~3.4	~7.7	~8.3
